# Wideband Fully-Programmable Dual-Mode CMOS Analogue Front-End for Electrical Impedance Spectroscopy

**DOI:** 10.3390/s16081159

**Published:** 2016-07-25

**Authors:** Virgilio Valente, Andreas Demosthenous

**Affiliations:** Department of Electronic and Electrical Engineering, University College London, WC1E 7JE London, UK

**Keywords:** analogue readout, electrical impedance spectroscopy (EIS), integrated circuits

## Abstract

This paper presents a multi-channel dual-mode CMOS analogue front-end (AFE) for electrochemical and bioimpedance analysis. Current-mode and voltage-mode readouts, integrated on the same chip, can provide an adaptable platform to correlate single-cell biosensor studies with large-scale tissue or organ analysis for real-time cancer detection, imaging and characterization. The chip, implemented in a 180-nm CMOS technology, combines two current-readout (CR) channels and four voltage-readout (VR) channels suitable for both bipolar and tetrapolar electrical impedance spectroscopy (EIS) analysis. Each VR channel occupies an area of 0.48 mm ${}^{2}$ , is capable of an operational bandwidth of 8 MHz and a linear gain in the range between −6 dB and 42 dB. The gain of the CR channel can be set to 10 kΩ, 50 kΩ or 100 kΩ and is capable of 80-dB dynamic range, with a very linear response for input currents between 10 nA and 100 μ A. Each CR channel occupies an area of 0.21 mm ${}^{2}$ . The chip consumes between 530 μ A and 690 μ A per channel and operates from a 1.8-V supply. The chip was used to measure the impedance of capacitive interdigitated electrodes in saline solution. Measurements show close matching with results obtained using a commercial impedance analyser. The chip will be part of a fully flexible and configurable fully-integrated dual-mode EIS system for impedance sensors and bioimpedance analysis.

## 1. Introduction

Electrical impedance spectroscopy (EIS) has recently gained widespread interest in electrochemical and biomedical research as a tool to study the electrical and physical properties of biological interfaces and to provide valuable diagnostic information about potential pathological conditions of biological cells, tissues and organs. EIS is applied to the measurement of tissue impedance for disease and cancer detection [1,2,3,4] and single-cell analysis [5,6] or used for imaging purposes, in electrical impedance tomography [7]. In the field of electrochemical analysis, EIS can be employed to interrogate functionalized biosensors to detect changes in impedance due to the presence of target analytes, including enzymes, antibodies and DNA [8,9,10], and to perform impedance microbiology [11].

In impedance microbiology, changes in the impedance of an electrochemical cell, formed by a set of microelectrodes immersed in a solution, is measured over time. These changes are due to the cell ionic metabolism, which directly affects the solution conductivity [12,13,14]. Most impedance methods measure only the conductivity of the medium using a pair of electrodes at a fixed frequency using a bipolar configuration. However, the measured impedance is the result of contributions from both the medium impedance and the electrode/interface impedance [15].

The contributions of the electrode and medium impedances are separated by changing the interrogation frequency, where the electrode impedance is predominant at low frequencies (~100 Hz), whereas the medium impedance is more significant at high frequencies (>10 kHz) [15]. The frequency range, however, depends greatly on the size and dimension of the interdigitated electrodes (IDEs) used for the measurements [11].

In addition, conventional impedance microbiology is not suitable for systems where bacterial concentration is determined by measuring the degradation of pH-sensitive polymer coatings of IDEs, which appears as a capacitance change over time [16]. In such cases, a single equivalent model cannot be used to describe the binding process, and the extraction of medium conductivity from the cell impedance profile becomes more complex. Moreover, the IDE capacitance is usually extracted at frequencies of kHz [16], thus increasing the frequency at which the medium conductivity is the sole contribution to the overall impedance.

In order to increase the flexibility of the impedance measurement, dual-mode EIS, based on both bipolar and tetrapolar configurations, can be exploited to measure the medium conductivity independently of the electrode impedance [17,18,19]. The use of two dedicated readouts for electrode and medium impedance measurement, however, puts a serious constraint on the cost-effectiveness and size of this type of impedimetric device, if targeted for point-of-care, wearable or implanted systems. Full integration of multichannel dual-mode readouts can provide low-cost solutions and potentially eliminate set-up time and calibration efforts required for separate units. Conventional EIS systems are designed for either voltage-mode measurements [17,20,21] or current-mode measurements [22,23,24]. Previous efforts in the design of flexible dual-mode systems are limited to the use of discrete units [25,26] or have been designed to detect small changes in solution or electrode impedance [27]. This paper presents the design of a multi-channel dual-mode analogue front-end (AFE) for EIS and bioimpedance analysis, which integrates two readout units for both bipolar (current-mode) and tetrapolar (voltage-mode) measurements.

**Figure 1:**
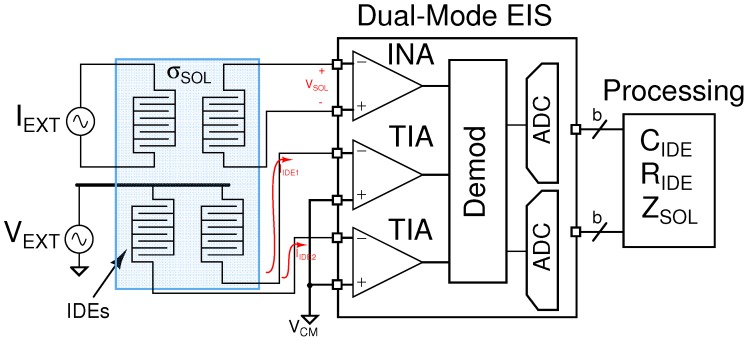
Conceptual arrangement of a dual-mode EIS measurement system with arrays of interdigitated electrodes (IDEs) to measure solution impedance and electrode/interface impedance. The solution impedance can be measured using a tetrapolar configuration by injecting a differential current, $I_{EXT}$ , through an electrode and measuring the differential voltage across a second electrode. Electrode/interface impedance can be measured by exciting an electrode with a voltage source, $V_{EXT}$ , and reading the current flowing through the electrode. In the example in the figure, the same external source is used on multiple electrodes.

**Figure 2:**
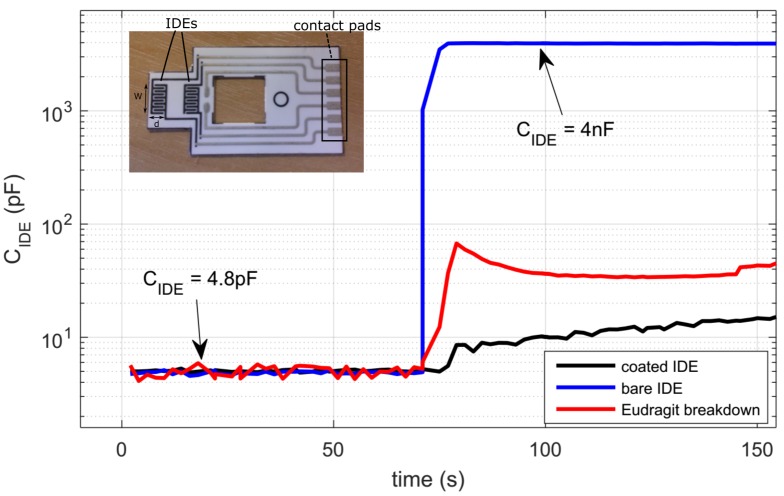
Example of measured capacitance over time of an IDE test strip shown in the figure inset. The average values of the capacitance are reported in the text for different types of IDEs. For convenience, only the first 150 s of measurement are shown. The values reported in the text refer to the steady state value after 1800 s. Each IDE test strip consists of two equal IDEs with an overall area (W × d) of 3 × 5.5 mm ${}^{2}$ . Each IDE consists of five digits of a length of 2 mm, a width of 0.3 mm and a spacing of 0.3 mm. The distance between the IDEs is 3 mm.

Figure 1 shows the concept of a dual-mode EIS system, where an array of IDEs is interrogated by both a voltage source and current source for both bipolar and tetrapolar measurements. In the former case, an AC voltage is applied across two terminals of one or more electrodes, and a transimpedance amplifier (TIA) is used to bias the electrode to a desired DC potential and to convert the IDE current into a voltage. The real and imaginary parts of the electrode admittance can then be extracted using a demodulator and converted into digital signals by an analogue-to-digital converter (ADC) for further processing of the electrode capacitive and resistive components. In the latter case, a differential current is applied through two terminals of one IDE, and differential voltages are measured by different IDEs, placed at different locations within the sample solution, using instrumentation amplifiers (INAs). This allows for the conductivity of the solution to be determined. The proposed integrated AFE is designed to interface with a set of carbon screen-printed IDEs coated with a pH-sensitive polymer (Eudragit S100) and shown in the inset of Figure 2. The impedance of the electrode shows nearly pure capacitive behaviour at frequencies around 20 kHz [16], thus allowing one to relate the change in capacitance of the electrode (from fully-coated to a degree of degradation) during a binding process to the concentration of the target analytes. The change of capacitance has been quantified (Figure 2) during wet experiments to a range of four orders of magnitude between a few pF to a few nF [16]. At 20 kHz the impedance of the IDE ranges approximately between 1 kΩ and 500 kΩ–1 MΩ. By using an excitation voltage of 10 mV, the resulting electrode current ranges between 10–20 nA and 10 μ A. The capacitance of the dry electrode ranges between 2 pF and 5 pF.

Although full characterization of the IDEs is beyond the scope of this paper, the performance of the AFE has been tested by measuring the capacitance of dry IDEs, the impedance of the electrodes in different buffer solutions and, concurrently, the conductivity of different solutions.

## 2. Analogue Front-End Chip

**Figure 3:**
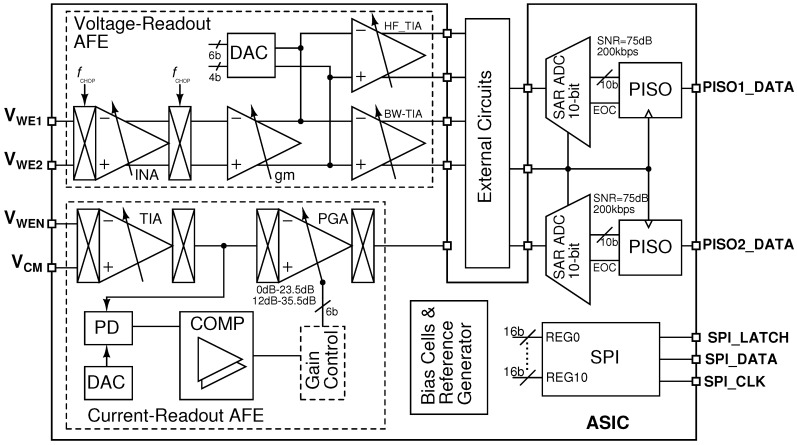
Chip architecture.

The architecture of the EIS AFE chip is shown in Figure 3. The chip allows for fully-differential voltage readout (VR, four channels) and single-ended rail-to-rail output current readout (CR, two channels). The CR channels are equipped with an automatic gain control (AGC) unit that allows extension of the signal dynamic range. The AGC comprises a peak detector (PD), a comparison stage (COMP) and a control logic unit (off-chip). In the VR mode, bandwidth-limited (BW) low-noise operation (up to 80 kHz) or high-frequency recording (HF, up to 8 MHz) can be selected. The BW channel can be used when extracellular voltage recording is needed in a frequency range from sub-Hz to kHz [28]. In this case, chopper-stabilized op-amps switching at a frequency of 100 kHz are used to allow for low-noise measurements to be performed. The HF channel allows for electrochemical sensing and solution conductivity mapping at frequencies higher than 10 kHz and up to 1 MHz [11]. Impedance measurements in the MHz regime are more sensitive to intracellular changes and can be used to characterize the cell membrane impedance [29]. Each channel has programmable gain stages, operational bandwidth and enable signals that can turn off entire sections of the chip to save power. The chip is powered by a single 1.8-V supply. The common-mode voltage, $V_{CM}$ , is set to 0.9 V by an internal voltage generator. The chip can be programmed via a microcontroller using a serial-to-peripheral interface (SPI) consisting of 11 registers. The chip is also equipped with two 10-bit successive-approximation ADCs with a maximum sampling rate of 200 kS/s. The output of the ADCs can be read serially though an on-chip parallel-in-serial-out (PISO) interface. The analogue outputs of the VR and CR units are directly available externally and can be readily connected to the on-chip ADCs for digitization. The operation of the chip described in this paper was limited to spectroscopy studies in a frequency range between 100 Hz and 100 kHz.

### 2.1. Current-Readout AFE

**Figure 4:**
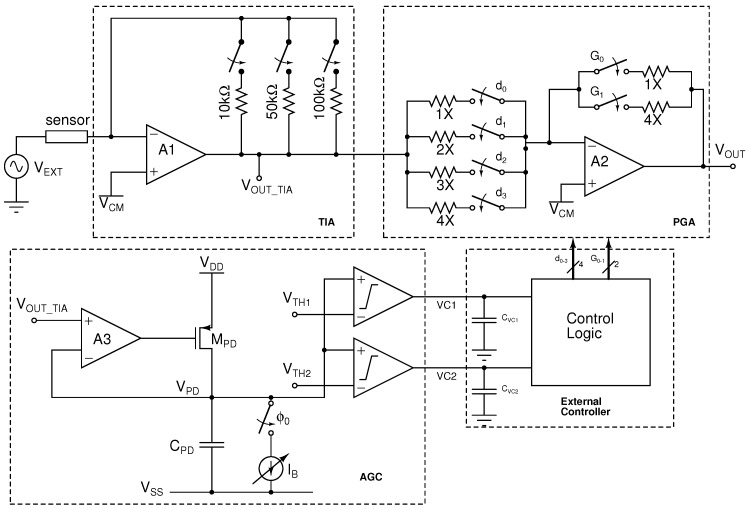
Architecture of the current-mode channel with automatic gain compensation.

The CR-AFE consists of a TIA input stage followed by a programmable gain amplifier (PGA) stage controlled by an AGC unit, as shown in Figure 4. The TIA can be implemented using either discrete-time (DT) or continuous-time (CT) architectures. DT topologies offer very low-noise performance, but are limited to low frequency operation due to the need to reset the integrating capacitor [30,31,32,33]. Among CT topologies, integrator-differentiator (ID) and resistor-feedback (RF) architectures are suitable candidates to implement TIAs in CMOS [34,35]. ID structures have several advantages, including low-noise and high-bandwidth operation [36], but require the implementation of very large resistors or DC-feedback loops to provide a DC current path, which introduces complexity and stability issues.

The TIA in this paper was implemented by a resistor-feedback op-amp configuration. This offers a straightforward implementation that can handle DC currents. In addition, input currents in the range of nA– μ A, consistent with the sensors used in this study, require small TIA gains (kΩ) to avoid the saturation of the amplifier, which can be better implemented using resistors. The gain of the TIA is programmable by switching the feedback resistor to 10 kΩ, 50 kΩ or 100 kΩ. A resistor-feedback programmable-gain amplifier is used to perform further amplification between 1 V/V and 14 V/V using $G_{0}$ and 4 V/V and 54 V/V using switch $G_{1}$ . Fine gain tuning is controlled by changing the input resistance of the amplifier, $R_{1}$ , so that the PGA gain is given by:

(1) $$A_{PGA}=-\frac{R_{2}}{R_{1}}=-\left(G_{0}R_{u}+4G_{1}R_{u}\right)\cdot \sum_{n=0}^{3}\frac{d_{n}}{2^{n}R_{u}}$$

where $R_{2}$ is the feedback resistance and $R_{u}$ is the value of the unit resistor of the array, *d* is the control code of the input resistor array and *n* is the number of bits and is equal to four.

**Figure 5:**
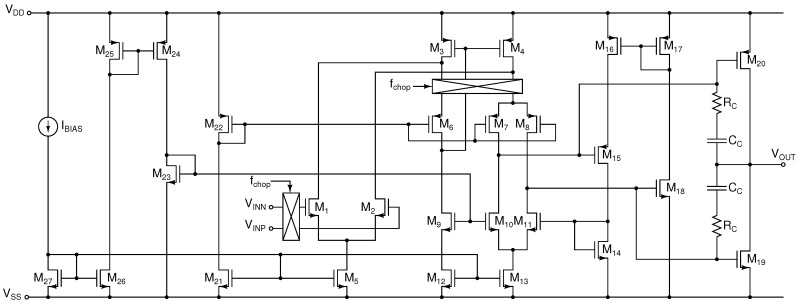
Schematic of the Class AB op-amp used to implement the TIA and the programmable gain amplifier (PGA).

Amplifiers A1 and A2 in Figure 4 are implemented by a two-stage Class AB amplifier with a minimum-current selector for high drive capability, rail-to-rail output and high-efficiency [37]. A simplified schematic of the Class AB amplifier is shown in Figure 5. The amplifier achieves a simulated DC gain of 120 dB, a unity gain bandwidth of approximately 34 MHz with a phase margin of 60° and consumes 105 μ A. The simulated input-referred noise is 98 nV/ $\sqrt{Hz}$ at 10 kHz and 35 nV/ $\sqrt{Hz}$ at 100 kHz.

#### 2.1.1. TIA Stability

**Figure 6:**
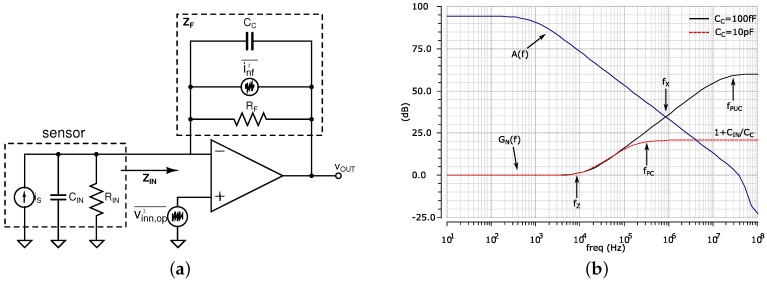
(**a**) Schematic of a TIA with the sensor model and noise sources; (**b**) simulated open-loop gain of the TIA op-amp and noise gain with different values of the compensation capacitor, $C_{C}$ .

The architecture of a TIA is shown in Figure 6. The sensor is modelled by a current source in parallel with a capacitance, $C_{IN}$ , and resistance, $R_{IN}$ , which represent the total impedance seen at the input of the amplifier. The feedback resistor $R_{F}$ determines the closed-loop DC gain of the amplifier, and the capacitor $C_{C}$ is used for compensation, as described in the following section. The noise sources associated with the TIA are modelled as a noise current source, $i_{nf}^{2}$ , associated with the feedback resistor, and a noise voltage source, $v_{inn,op}^{2}$ , that represents the noise in the amplifier devices.

The TIA transfer function is given by:

(2) $$\begin{array}{c} G_{TIA}\left(f\right)=\frac{v_{OUT}}{i_{s}}\left(f\right)=-\frac{R_{F}}{1+j\frac{f}{f_{P}}} \end{array}$$

where $f_{P}$ = 1/(2*π* $R_{F}$ $C_{C}$ ) is the pole of the amplifier.

The stability of the amplifier depends on the magnitude of the total input capacitance, $C_{IN}$ , of the amplifier, which generates a zero in the noise gain, $G_{N}\left(f\right)$ , given by:

(3) $$\begin{array}{c} G_{N}\left(f\right)=\frac{1+j\omega R_{F}\left(C_{IN}+C_{C}\right)}{1+j\omega R_{F}C_{C}} \end{array}$$

In order to guarantee stability, the TIA needs to have enough phase margin for A(f) β(f) ≥ 1, where A(f) is the open-loop gain of the TIA op-amp and β(f) is the feedback factor, given by 1/ $G_{N}\left(f\right)$ . The intercept frequency between A(f) and $G_{N}\left(f\right)$ , $f_{X}$ , is then a critical point for stability analysis. Ensuring that the difference in the slopes between the two curves in ≤20 dB, the TIA will have enough phase margin and grant stability. The compensation capacitor, $C_{C}$ , can be adjusted in order to introduce a pole and flatten the noise gain response before the crossover frequency. Figure 6b shows simulated results of the A(f) and $G_{N}\left(f\right)$ for different values of $C_{C}$ of 100 fF and 10 pF, with $C_{IN}$ set to 100 pF and $R_{F}$ equal to 100 kΩ. If the capacitance is too small, the pole frequency, $f_{PUC}$ , will be beyond the intersect point and will cause instability. This will result in peaking in the closed-loop response of the TIA. $C_{C}$ can then be increased to shift the pole frequency, $f_{PC}$ , below $f_{X}$ . The frequency of the pole generated by $R_{F}$ and $C_{C}$ will set the overall bandwidth of the TIA.

#### 2.1.2. TIA Noise

The equivalent input-referred current noise of the TIA can be derived as the sum of the contributions of device noise from the amplifier devices and the noise of the feedback resistor, $R_{F}$ . The output voltage noise of the op-amp, $v_{on,amp}^{2}$ , can be derived with the amplifier configured as a non-inverting stage and is given by:

(4) $$\begin{array}{cc} v_{outn,op}^{2} & =v_{inn,op}^{2}{\left|1+\frac{Z_{F}}{Z_{IN}}\right|}^{2} \end{array}$$

where $v_{inn,op}^{2}$ is the op-amp input-referred voltage noise and $Z_{IN}$ and $Z_{F}$ are the input and feedback impedances, respectively.

Expanding Equation (4), with $R_{IN}$ >> $R_{F}$ , yields:

(5) $$\begin{array}{cc} v_{outn,op}^{2} & =v_{inn,op}^{2}\left[\frac{1+4\pi^{2}f^{2}R_{F}^{2}{\left(C_{C}+C_{IN}\right)}^{2}}{\left(1+4\pi^{2}f^{2}C_{C}^{2}R_{F}^{2}\right)}\right] \end{array}$$

The input-referred noise of the op-amp relating to $v_{outn,op}^{2}$ can then be derived from Equations (2) and (6) as:

(6)iinn,op2=voutn,op2GTIA2(7)=vinn,op21+4π2f2RF2(CC+CIN)2RF2

The total equivalent input-referred current noise of the TIA, $i_{n,eq}^{2}$ is then given by:

(8)in,eq2¯=inf2¯+iinn,op2¯(9)=4kTRF+vinn,op2¯1+4π2f2RF2(CC+CIN)2RF2

Equation (9) shows how the total equivalent noise of the TIA is inversely proportional to the feedback resistor, $R_{F}$ . Maximising $R_{F}$ , therefore, will decrease the input-referred noise at the cost, however, of the TIA bandwidth. The op-amp input-referred noise source, $v_{inn,op}^{2}$ , is given by:

(10) $$\begin{array}{cc} \bar{v_{inn,op}^{2}} & =\frac{K_{F}^{\prime }}{WLC_{OX}^{2}}\frac{1}{f}+\frac{16kT}{3g_{m}} \end{array}$$

where the first term in Equation (10) represents the low-frequency flicker (*1/f*) noise and the second terms represents the thermal noise. $K_{F}$ is the flicker noise coefficient and depends on the CMOS technology, with $K_{F}^{\prime }$ = $K_{F}/\mu$ , $C_{OX}$ is the device oxide capacitance and $g_{m}^{2}=\mu C_{OX}\left(W/L\right)I_{DS}$ is the device transconductance. As EIS is usually performed over a wide frequency range (e.g., 10 ${}^{2}$ Hz–10 ${}^{6}$ Hz), the frequency range over which the op-amp noise is dominated by either the *1/f* or the thermal noise components, can be identified by estimating the noise corner frequency, $f_{c}$ , when the frequency at which the asymptotes of the *1/f* and thermal noise components cross. This is determined from Equation (10) as:

(11) $$\begin{array}{cc} f_{c} & =\frac{6K_{F}I_{D}}{16kTg_{m}C_{OX}L^{2}} \end{array}$$

The device sizes and bias current of the op-amp can be selected to set $f_{c}$ below the lowest frequency of interest. Combining Equations (9) and (10), we can derive the total input-referred noise of the TIA as:

(12) $$\begin{array}{cc} \bar{i_{inn,t}^{2}} & =\frac{4kT}{R_{F}}+\left[\frac{K_{F}^{\prime }}{WLC_{OX}^{2}g_{m}}\frac{1}{f}+\frac{16kT}{3g_{m}^{2}}\right]\left[\frac{1+4\pi^{2}f^{2}R_{F}^{2}{\left(C_{C}+C_{IN}\right)}^{2}}{R_{F}^{2}}\right] \end{array}$$

Equation (12) shows that the overall TIA noise is inversely proportional to the feedback resistors, $R_{F}$ , and proportional to *1/f* at low frequencies (< $f_{c}$ ) and $f^{2}$ at high frequencies (≫ $f_{c}$ ).

#### 2.1.3. Automatic Gain Control

The AGC unit tracks the amplitude of the TIA output, $V_{OUT\_TIA}$ , by means of a PD. The PD consists of an error amplifier, A3 , a switch, $M_{PD}$ , a capacitor, $C_{PD}$ , and a discharge current source, $I_{B}$ . Amplifier A3 senses the difference between the output of the TIA, $V_{OUT\_TIA}$ , and the voltage of the PD capacitor, $V_{PD}$ . As the input signal increases, $M_{PD}$ is open, and $C_{PD}$ is charged. After the input signal reaches its peak value, it will start decreasing, causing the error amplifier to saturate, due to its high open-loop gain and turn off $M_{PD}$ . The peak amplitude of the input signal is held on $C_{PD}$ . As the input signal starts decreasing, $C_{PD}$ can be discharged passively, through the output impedance of the PD plus parasitic resistances, or actively, by shorting it to $V_{SS}$ . A full discharge of the track-and-hold capacitor is acceptable so long as the frequency of the input signal is low. If the frequency of the input signal is high, the peak detector may be too slow at tracking the input signal and charging up to the input signal peak value.

**Figure 7:**
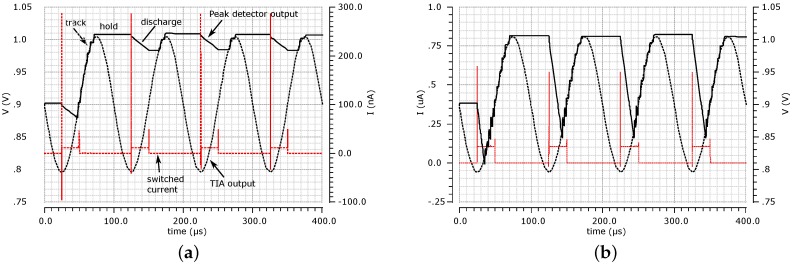
Transient simulation of the adaptive PD unit at 10 kHz. The relaxation current is set to (**a**) 10 nA and (**b**) 100 nA and switched at the same frequency as the input signal with a 25% duty cycle. The output capacitor of the PD (external) is 300 pF.

A novel active discharge strategy was employed, whereby the switched current source, $I_{B}$ , is used to ‘relax’ the capacitor after a peak is detected. $I_{B}$ is switched at the frequency of the input signal with variable duty cycle, resulting in three phases of operation. During the tracking phase, $\phi_{0}$ is open, and $V_{OUT\_TIA}$ is stored on $C_{PD}$ . Once the peak amplitude is reached, $V_{OUT\_TIA}$ is held on $C_{PD}$ . After a programmable delay, $C_{PD}$ can be actively discharged by $I_{B}$ . The transient behaviour of the adaptive PD is shown in Figure 7 for a 10-kHz input signal. The output capacitor is 300 pF, and the switch current is set to 10 nA (Figure 7a) and 100 nA (Figure 7b).

The benefit of this architecture over conventional ones is two-fold. Firstly, a large output capacitor can be used, which allows for a more accurate reading of the peak input voltage. In the conventional PD architecture, a trade-off exists between the value of the storing capacitor and the speed or tracking of the PD. Passive discharge limits the response to the PD signals, which rapidly change in amplitude. Secondly, by using a programmable discharge current, the behaviour of the PD can be made independent of the input frequency.

### 2.2. Voltage-Readout AFE

**Figure 8:**
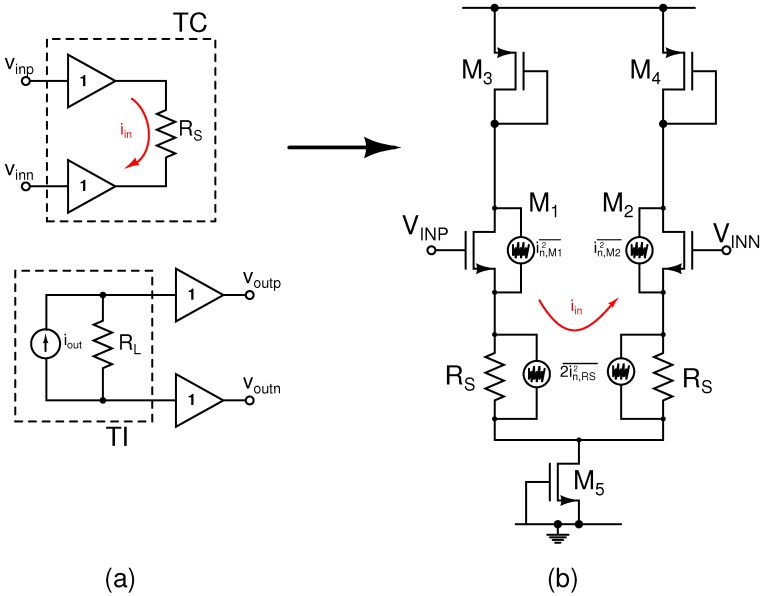
Current-mode amplifier model. (**a**) Architecture (**b**) schematic of the input TC with noise sources.

Each VR channel comprises two stages of fully-differential current-mode variable-gain amplifiers, which provide low-noise and high CMRR [38]. The architecture of a current-mode amplifier is shown in Figure 8a and consists of a transconductor (TC) stage and a transimpedance (TI) stage. The TC stage buffers the input voltage, which appears across the resistor, $R_{S}$ , and results in a current, $i_{in}$ , flowing in the input stage. This current is then copied to the output stage, $i_{out}$ , which flows through the load resistor, $R_{L}$ , and generates an output voltage $V_{out}$ with a gain determined by $R_{L}/R_{S}$ . The input TC dominates the noise profile of the current-mode amplifier. Figure 8b shows a schematic of the input TC with equivalent noise sources. The noise contributions of $M_{5}$ are negligible, as it appears as a common-mode signal cancelled by the differential stage. The noise of the current-mirror load, $M_{3}$ - $M_{4}$ , is assumed to be much smaller than the noise of the input pair as $g_{m3,4}$ << $g_{m1,2}$ .

The TC transfer function is given approximately by:

(13) $$\begin{array}{c} i_{out}=\frac{v_{in}}{\left(R_{S}+\frac{2}{g_{m1,2}}\right)} \end{array}$$

The total output noise current is given by the contributions of the input-pair transistors, $\bar{i_{nM}^{2}}$ and the source-degeneration resistors $\bar{i_{RS}^{2}}$ as:

(14)ioutn2¯=2inM1,22¯+2iRS2¯(15)=163kTgm1,2+KFCOXWL1f+8kTRS

The total input-referred noise is then determined by Equations (13) and (15) as:

(16) $$\begin{array}{cc} \bar{v_{inn}^{2}} & =2\bar{i_{outn}^{2}}{\left(R_{S}+\frac{2}{g_{m1,2}}\right)}^{2} \end{array}$$

Assuming $g_{m}$ $R_{S}>>$ 1, Equation (16) becomes:

(17) $$\begin{array}{cc} \bar{v_{inn}^{2}} & =R_{S}\left[\frac{16}{3}kTg_{m1,2}R_{S}+\frac{K_{F}R_{S}}{C_{OX}WL}\frac{1}{f}+8kT\right] \end{array}$$

The TC input-referred thermal noise is then given by:

(18) $$\begin{array}{cc} \bar{v_{inn,th}^{2}} & =8kT\left(1+\frac{2}{3}g_{m1,2}R_{S}\right) \end{array}$$

Note that a series degeneration resistance was preferred over a shunt topology. If the latter is chosen, the noise contribution of the tail current sources will no longer be negligible, and the total input-referred thermal noise will be increased by a factor of $4kT\left(2/3\right)g_{mS}$ , where $g_{mS}$ is the transconductance of the tail current source (equivalent to $M_{5}$ in Figure 8b).

**Figure 9:**
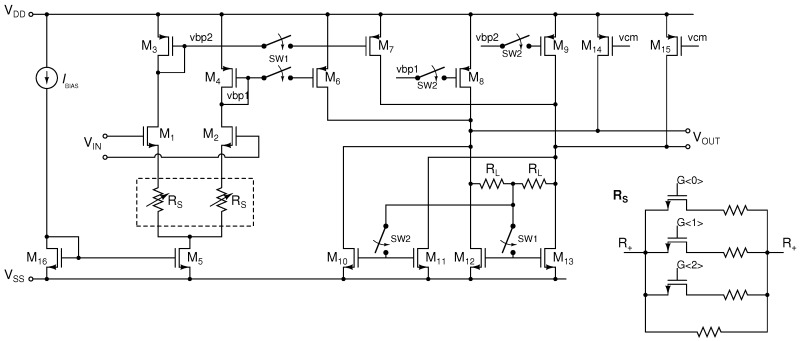
Schematic of the VR AFE INA.

The first stage buffer architecture is shown in Figure 9. The input pair formed by $M_{1}$ and $M_{2}$ is degenerated by a source resistor, $R_{S}$ , resulting in an AC current equal to the ratio between the input voltage, $V_{IN}$ , and the sum of the source resistance of the input pair (= 1/2 $g_{m}$ ) and $R_{S}$ .

The current is copied to the output by load transistor $M_{3}$ and $M_{4}$ . The gain of the amplifier is proportional to the ratio of the load resistor, $R_{L}$ and $R_{S}$ .

By switching $R_{S}$ , instead of $R_{L}$ , the amplifier gain can be varied while keeping its bandwidth approximately constant. $R_{S}$ is implemented by a three-bit resistor array, shown in the inset of Figure 9. $R_{L}$ is set to 2.1 kΩ, and the unit resistor of the bank is set to 3.5 kΩ. Extensive simulations were performed to optimized the sizing of the resistor bank to obtain accurate gain values.

A common-mode feedback (CMFB) circuit was implemented in order to stabilize the output common mode voltage to mid-rail (0.9 V). This is based on a difference-differential amplifier that senses the differential output and injects common-mode current to the amplifier output via current sources $M_{14}$ and $M_{15}$ . The sink transistors $M_{10}$ – $M_{13}$ are scaled to provide a larger current than the source transistors, $M_{6}$ – $M_{9}$ , in order to make the CMFB effective. The sink transistors are also switched depending on the output current settings SW1 and SW2 in order to save power when the low gain setting is selected. The gain range can be selected by controlling switches SW1 and SW2 in Figure 9.

The buffer is biased with a tail current source of 120 μ A, resulting in the input pair source resistance, $1/g_{m}$ , of 700 Ω. The output current through each branch is switched between 60 μ A and 300 μ A. The on-resistance of the switches was designed to be in the order of 20 Ω, a factor of 10 smaller than the lowest value of $R_{S}$ , which is set to 200 Ω.

**Figure 10:**
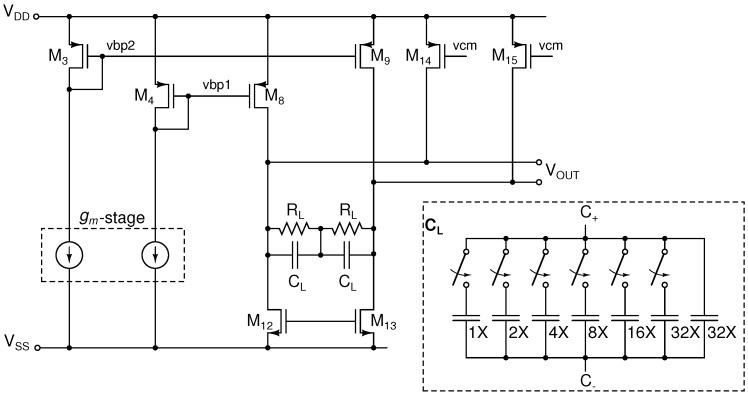
Schematic of the BW-limited TIA stage of the INA.

The second stage buffer of the VR AFE is split into a high-frequency channel (HF) and a bandwidth-limited (BW) channel. Both channels share a common $g_{m}$ -stage, which is equivalent to the $g_{m}$ -stage of the first-stage buffer. The output stage TIA of the BW channel, shown in Figure 10, is loaded by a tunable capacitor, $C_{L}$ , which allows one to select the amplifier bandwidth between 30 kHz and 80 kHz with a six-bit resolution. The capacitor array consists of 500 fF unit capacitors. The BW channel was designed to have a high gain between 12 dB and 42 dB, whereas the HF channel gain ranges between −6 dB and 22 dB.

Due to the large gain range that the VR AFE provides, an offset trimming unit was included. This is based on the use of current-steering DACs, which unbalance the current in the $g_{m}$ -stage input pair, in order to reduce the output offset voltage to zero. A four-bit DAC biased with a 750-nA current is used for offset coarse calibration, and a six-bit DAC biased with a 10-nA current is used for fine calibration. The offset calibration unit is designed to compensate for input offsets as high as ±20 mV.

## 3. Measurements

### 3.1. CR AFE

**Figure 11:**
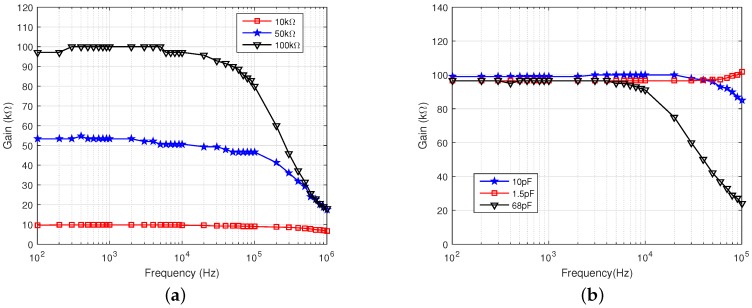
Measured frequency response of the TIA for (**a**) different gain settings and (**b**) different feedback capacitor values at a gain of 100 kΩ.

Figure 11 shows the frequency response of the CR AFE TIA in a frequency range between 100 Hz and 1 MHz. The gain of the TIA was measured to be 10 kΩ, 50 kΩ and 100 kΩ (Figure 11a). The bandwidth of the TIA was measured to be 1.59 MHz, 310 kHz and 159 kHz, respectively, when using a feedback capacitor of 10 pF in all cases. The measurements agree with the theoretical bandwidth of the TIA given by $1/(2\pi R_{F}C_{F})$ . Figure 11b shows the TIA frequency response at a gain of 100 kHz for different feedback capacitors (with values reported in the figure legend). The minimum value of the feedback capacitor is set to 10 pF in order to avoid the occurrence of unwanted gain increase (gain peaking) at high frequencies, as shown in the figure for a capacitor value of 1.5 pF.

**Figure 12:**
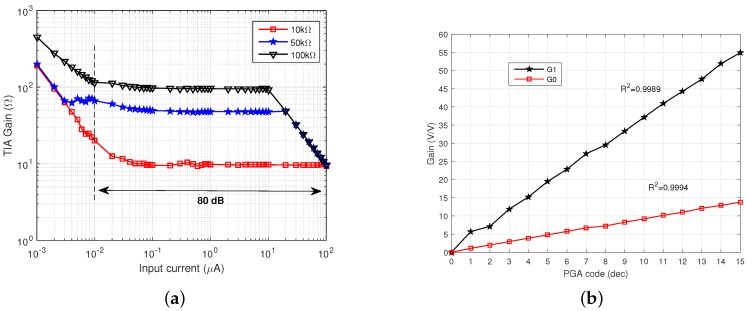
Measured range and linearity of the (**a**) TIA and (**b**) PGA.

The linearity of the TIA was measured by sweeping an input test current source between 1 nA and 100 μ A at a frequency of 10 kHz. The dynamic range of the TIA is shown in Figure 12a at different gains. The linear range was measured between the minimum and maximum input current, which resulted in a deviation of the gain larger than 1 dB from its nominal value. This range was measured to be 100 nA–100 μ A for a gain of 10 kΩ, 50 nA–20 μ A for a gain of 50 kΩ and 10 nA–1 μ A for a gain of 100 kΩ, resulting in an overall linear dynamic range between 10 nA and 100 μ A, or 80 dB.

The gain and linearity of the PGA were measured by setting the TIA input current to 1 μ A and the TIA gain to 10 kΩ, and they are shown in Figure 12b. The gain of the PGA was measured in a range between 1 and 13.8 V/V (0–23 dB) for gain setting $G_{0}$ and 5.7 and 55 V/V (15–35 dB) for gain setting $G_{1}$ .

**Figure 13:**
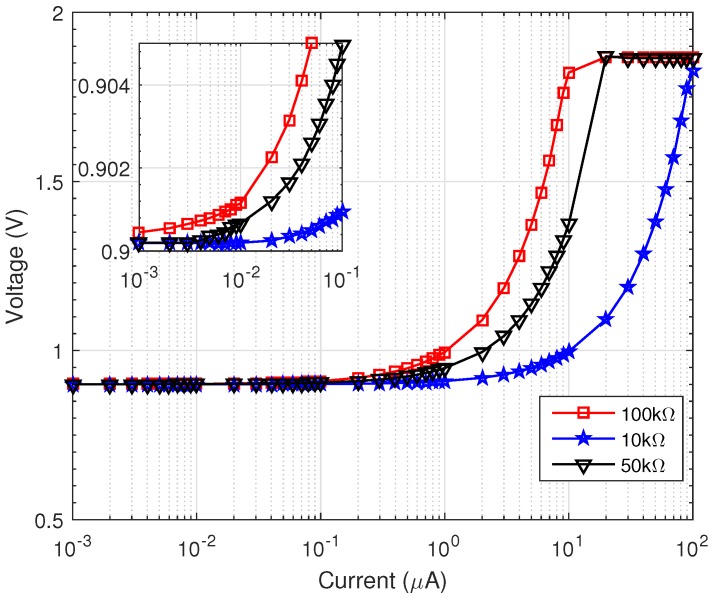
Measured output range of the PD versus TIA input current, with an external capacitor of 300 pF. Inset: Output profile for an input current range between 1 nA and 100 μ A.

The performance of the PD was measured in the time-domain, as the same time as the measurements of the performance of the TIA reported above. In order to measure the output range of the PD, an external 1-nF capacitor was connected to the output of the PD and $I_{B}$ was switched off. The output DC voltage was measured with a multimeter for input currents varying between 1 nA and 100 μ A and is reported in Figure 13. The figure shows that the PD dynamic range extends from 10 nA–100 μ A, which is consistent with the TIA linear range.

**Figure 14:**
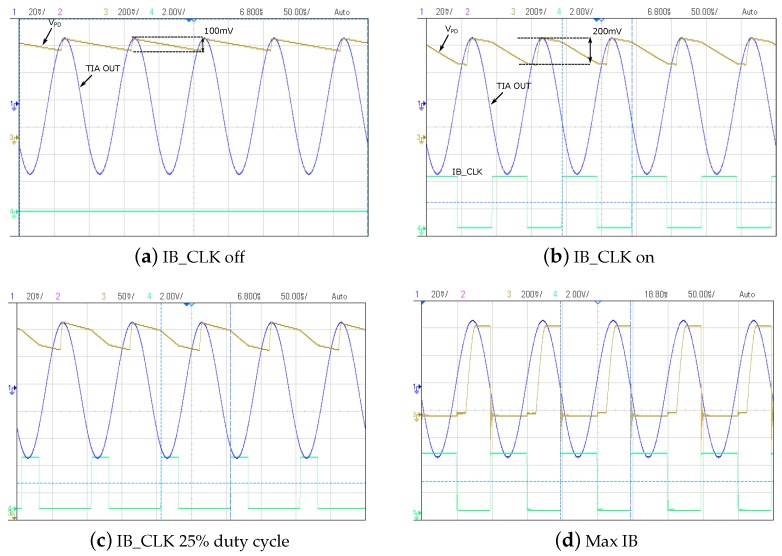
Real-time operation of the peak detector.

Figure 14 shows the real-time operation of the PD under different conditions in response to a 1- μ A input current at a frequency of 10 kHz. In the case shown in Figure 14a, $I_{B}$ is off, and $V_{PD}$ tracks the input signal, while this is increased and passively discharges after the input peak amplitude has been reached. In the cases reported in Figure 14b,c, $I_{B}$ is switched at the frequency of the input signal with a duty cycle of 50% and 25%, respectively. The three operating phases described in Section 2.1 are clearly visible. After the tracking phase, the peak amplitude of the input signal is held on $V_{PD}$ as long as $I_{B}$ is off and can, therefore, be accurately sampled during this phase. As $I_{B}$ is turned on, $C_{PD}$ is discharged to a level that depends on the amplitude of $I_{B}$ . By varying the duty cycle of IB_CLK , the duration of two sampling phases (before and after the capacitor discharging) can be controlled. Finally, the PD can be forced to behave equivalently to shorting $C_{PD}$ to $V_{SS}$ by further increasing the amplitude of $I_{B}$ as shown in Figure 14b.

### 3.2. VR AFE

The frequency response of the VR AFE was measured between 100 Hz and 10 MHz by applying a 100-mV fully differential input signal with a DC offset of 0.9 V. The bandwidth of the BW channel was measured by varying the value of the load capacitance. A range between 30 kHz and 80 kHz is achievable in steps of approximately 800 Hz.

**Figure 15:**
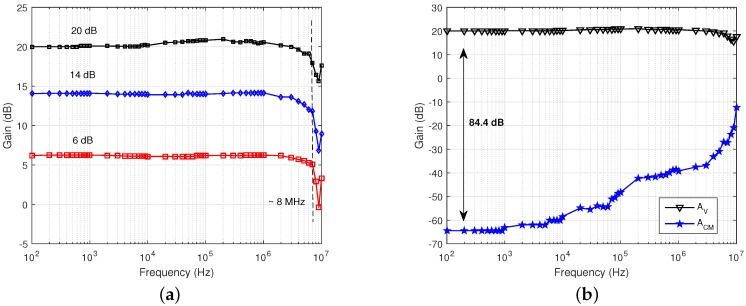
Measured (**a**) frequency response and (**b**) CMRR at 20 dB of the INA.

The frequency response of the HF channel is shown in Figure 15a for different gain settings. The bandwidth of the buffer was measured to be approximately 8 MHz and is substantially independent of the amplifier gain. The amplifier measured CMRR is shown in Figure 15b for a gain of 20 dB. At low frequencies below 1 kHz, the CMRR is greater than 84 dB.

**Figure 16:**
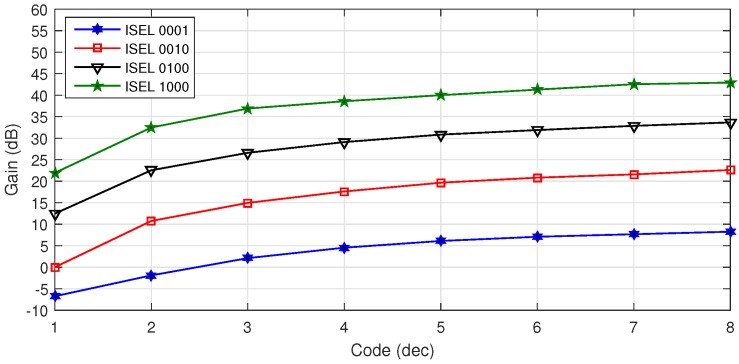
Measured VR AFE gain range for different gain settings.

The gain range and linearity of the VR AFE is reported in Figure 16 for both BW and HF channels. The overall gain can be set between −6 dB and 22 dB for the HF channel and between 12 dB and 42 dB for the BW channel.

The noise of the TIA and INA was measured using a spectrum analyser (MDO41046) within a bandwidth between 1 kHz and 1 MHz and a resolution bandwidth of 1 kHz. The inputs were grounded, and the gains were set to the maximum value (100 kΩ for the TIA and 42 dB for the INA). The noise floor of the TIA was measured at 100 kHz to be 450 fA/ $\sqrt{Hz}$ , and the noise floor of the INA was measured to be 10 nV/ $\sqrt{Hz}$ .

**Figure 17:**
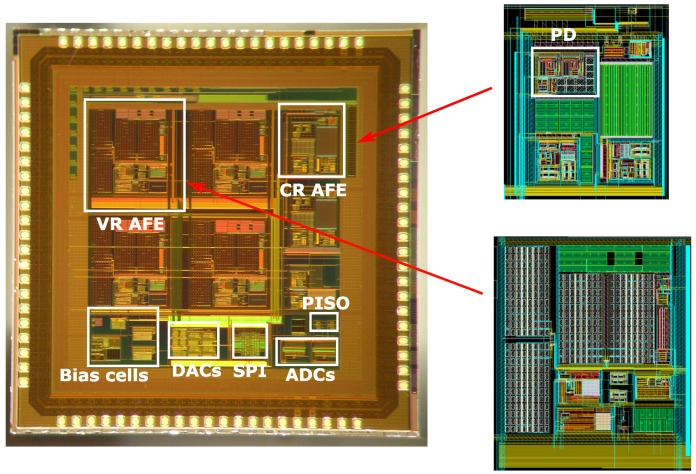
EIS AFE chip microphotograph.

Figure 17 shows a microphotograph of the chip implemented in a 0.18- μ m CMOS process. The chip includes 84 pads and has a total area of 10 mm ${}^{2}$ . The active area is 4.2 mm ${}^{2}$ . The measured performance of the chip is summarized in Table 1.

### 3.3. Impedance Measurements

The chip was interfaced to a set of IDEs in different aqueous solutions. A set of IDEs consisted of bare carbon interdigitated screen-printed electrodes. Another set of IDEs was coated with pH-sensitive polymer (Eudragit S 100).

The IDEs were excited by a small AC signal. Voltage and current recordings were stored on a computer and coupled with a digital signal processing software unit (MATLAB) to extract impedance values for a range of frequencies between 100 Hz and 100 kHz (10 points per decade). The VR AFE HF channel was used in these experiments to measure the voltage profiles in the solutions. Chopper stabilization was not enabled during this set of experiments, due to the frequency range of the measurements and the measured current and voltage levels. The chopping switches were disabled by tying their gates to the supplies. The upper limit of 100 kHz was set by the maximum sampling rate of the on-chip 10-bit ADC of 200 kS/s.

System calibration was performed by measuring the impedance magnitude and phase of known resistor and capacitor values and correcting for gain and phase factors during post-processing.

**Figure 18:**
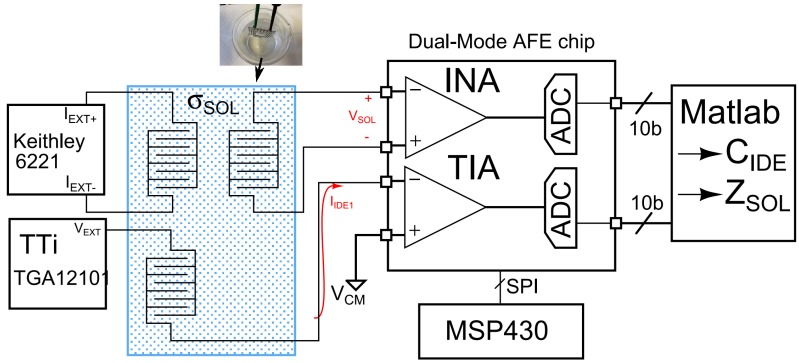
Experimental set-up. Two sets of IDEs were used in the experiments to measure the interface impedance and the solution conductivity, which was changed by varying the concentration of NaCl.

The experimental set-up is shown in Figure 18. One IDE was excited with an AC voltage of an amplitude between 10 mV and 100 mV, and the current, $I_{IDE}$ , was measured using the CR channel of the chip. A tetrapolar configuration was used to measure the voltage profiles in the solution in response to a differential current, $I_{EXT}$ , of 1 mA.

**Figure 19:**
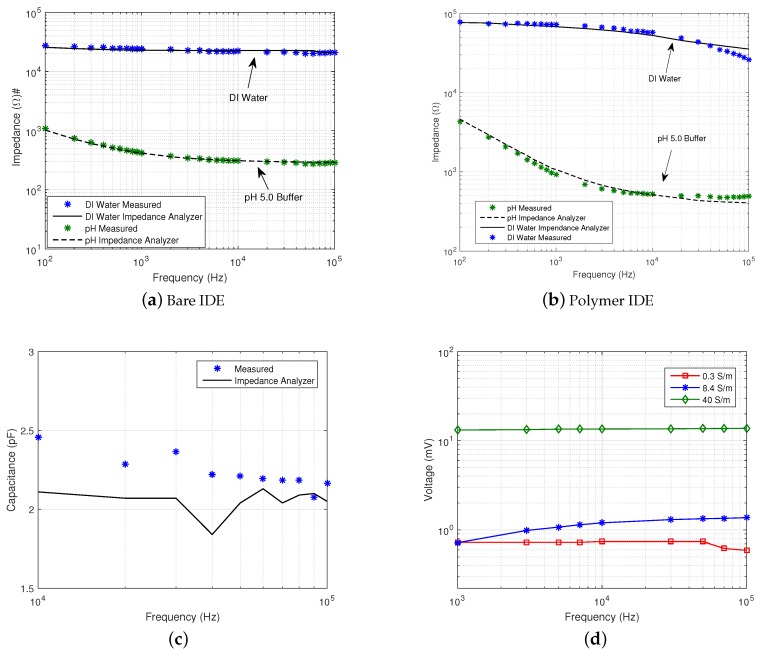
Impedance measurements. (**a**) Impedance of a bare IDE and (**b**) a polymer-coated IDE in DI water and pH 5.0 buffer solution; (**c**) capacitance of a dry IDE versus frequency and (**d**) tetrapolar voltage measurements in saline solutions of different conductivities.

Impedance spectra of bare and polymer-coated IDEs in DI water and pH 5.0 buffer solution were measured and compared to recordings obtained using a commercial impedance analyser (Wayne Kerr 6220B). The excitation voltage was set to 10 mV, and the gain of the CR AFE was set to 100 kΩ, by observing the lowest error compared to lower gains. Results are shown in Figure 19a,b.

The measurements show close matching between the impedance points recorded with the chip and the commercial system. The maximum discrepancies between the bare IDE cell measurements were 6% a 100 Hz and 9% at 400 Hz in pH 5.0 and DI water, respectively. For the polymer IDE cell, the largest error was estimated to be 17.4% at 100 kHz and 20.2% at 500 Hz pH 5.0 and DI water, respectively. The large error at low frequencies can be attributed to the *1/f* noise of the front-end, whereas the error at high frequencies is due to the bandwidth of the CR AFE, which was set to 159 kHz. Within the frequency range of 10 kHz–100 kHz, measurement accuracy is within 5% with errors as low as 0.2% in the case of bare IDE in pH 5.0 buffer solution.

The capacitance of the bare IDE was extracted by measuring the impedance of the dry electrode within a frequency range between 10 kHz and 100 kHz, at which the electrode shows nearly pure capacitive behaviour. Figure 19c shows the measured extracted capacitance. The average measured capacitance across the frequency range was 2.05 pF, closely matching the average capacitance of 2.23 pF measured with the impedance analyser. Figure 19d shows measured voltages in saline solutions with different conductivities using the tetrapolar configuration in Figure 18c. The conductivity of the solution was changed by adding different concentrations of NaCl and measured with a conductivity meter (with values reported in the figure). A differential 1-mA current was applied to one IDE, and the voltage, $V_{SOL}$ , was measured differentially across the second IDE. The resulting voltages at 10 kHz were measure to be 0.74 mV, 1.2 mV and 13.5 mV for conductivities of 0.3 S/m, 8.4 S/m and 40 S/m, respectively. The ratio between $I_{EXT}$ and $V_{SOL}$ was calculated with values measured at a frequency of 10 kHz and resulted in extracted conductivity values of 0.34 S/m 8.8 S/m and 37 S/m, respectively. Figure 19 shows how the chip is capable of concurrent measurement of both IDE impedance and solution conductivities.

A comparison between the presented system and a number of AFEs developed for EIS is shown in Table 2. The system reported in this work compares favourably in terms of configurability, gain and frequency range and power consumption.

## 4. Conclusions

This paper has presented the design and measured performance of a dual-mode analogue front-end chip, capable of parallel measurement of multi-channel current and voltage profiles for EIS. The adoption of the dual-mode EIS microsystem can be beneficial in the field of impedance microbiology by enabling separate measurements of the electrode/interface impedance and the medium impedance, as an alternative to conventional methods of determining impedance contributions by changing the interrogation frequency [15]. A dual-mode EIS can also be potentially employed to measure cellular behaviour at the electrode interface and at a distance from the surface of the electrodes [39] and to generate cellular impedance tomographic maps [28]. This system compares favourably to previous dual-mode systems [25,26,27]. It achieves high linearity, wideband operation and wide dynamic range and provides a potential cost-effective interface for several EIS applications. The voltage sensing unit provides very wide operation bandwidth up to 8 MHz and a large linear gain range from −6 dB of attenuation to 42 dB of amplification, achieving a CMRR greater than 80 dB.

The current sensing unit provides 80 dB of linear range and a current detection limit as low as 10 nA with configurable gain and bandwidth settings. The flexibility is further enhanced by a dynamic peak detector unit, which can be operated within an automatic gain control loop to optimize the channel dynamic range under different measurement conditions. The use of small values for the feedback resistor in the TIA (Figure 4) was required to accommodate large currents flowing through the IDE (>100 nA). This, however, results in larger input-referred current noise as compared to alternative structures [33,35]. Nevertheless, given the relatively large input currents, a more power-efficient architecture was preferred. The authors are further developing the system toward a fully-integrated dual-mode EIS platform, comprised of a multi-channel dual-mode arbitrary waveform generator, a dual-mode analogue readout interface and a digital impedance processing unit [40].

## Figures and Tables

**Table 1:** Summary of chip measured performance.

PARAMETER	UNITS	VALUE
Technology	-	0.18 μ m CMOS
Number of pads	-	84
Chip active area	mm ${}^{2}$	4.2
Supply voltage	V	1.8
**VR AFE**		
Gain range	dB	−6 to 42
BW LF	kHz	30 to 80
BW HF	MHz	8
CMRR	dB	84.4
Max current consumption	μ A	690
**CR AFE**		
TIA gain	kΩ	10, 50, 100
TIA dynamic range	dB	80
PGA gain	V/V	1 to 55
Max current consumption	μ A	530

**Table 2:** Performance comparison of the analogue front-end for EIS.

PARAMETER	CH	This Work	[35]	[33]	[21]	[4]
Technology		0.18 μ m CMOS	0.35 μ m CMOS	0.35 μ m CMOS	0.18 μ m CMOS	0.18 μ m CMOS
Supply voltage (V)		1.8	3.3	3.3	5	1.8
Readout modality		Dual-mode	Current	Current	Voltage	Voltage
Gain (kO)	CR	10, 50, 100	60,000	-	-	-
	VR	−6 dB–42 dB	-	-	15 dB–32 dB	18 dB–60 dB
Bandwidth (Hz)	CR	100 k–1.59 M	4 M	4k	-	-
	VR	8 M	-	-	76 k	100 k
Resolution (bits)		10	-	-	10	14
Power/channel (mW)	CR	1.24	55	23		-
	VR	0.95	-	-	6	53.4
$i_{inn}$ (fA/ $\sqrt{Hz}$ )	CR	450	4	5	-	-
$v_{inn}$ (nV/ $\sqrt{Hz}$ )	VR	10	-	-	-	36

^1^ Measured at 100 kHz with gain set to 100 kΩ; ^2^ measured at 100 kHz with gain set to 42 dB.

## Abbreviations

The following abbreviations are used in this manuscript:

IDEs: Interdigitated electrodes
EIS: Electrical impedance spectroscopy
AFE: Analogue front-end
VR: Voltage readout
CR: Current readout
TIA: Transimpedance amplifier
INA: Instrumentation amplifier
AGC: Automatic gain control
PD: Peak detector
SPI: Serial-to-peripheral interface
PISO: Parallel-in-serial-out
HF: High frequency
BW: Bandwidth limited
CMFB: Common-mode feedback
CMRR: Common-mode rejection ratio

## References

[1] Yufera A., Rueda A., Munoz J., Doldan R., Leger G., Rodriguez-Villegas E.O. (2005). A tissue impedance measurement chip for myocardial ischemia detection. IEEE Trans. Circuits Syst. I Regul. Pap..

[2] Halter R.J., Hartov A., Heaney J.A., Paulsen K.D., Schned A.R. (2007). Electrical impedance spectroscopy of the human prostate. IEEE Trans. Biomed. Eng..

[3] Constantinou L., Bayford R., Demosthenous A. (2015). A wideband low-distortion CMOS current driver for tissue impedance analysis. IEEE Trans. Circuits Syst. II Express Briefs.

[4] Hong S., Lee K., Ha U., Kim H., Lee Y., Kim Y., Yoo H.-J. (2015). A 4.9 mΩ-sensitivity mobile electrical impedance tomography IC for early breast-cancer Detection System. IEEE J. Solid State Circuits.

[5] Cho Y.C.Y., Kim H.S.K.H.S., Frazier A.B., Chen Z.G., Shin D.M.S.D.M., Han A. (2009). Whole-cell impedance analysis for highly and poorly metastatic cancer cells. J. Microelectromech. Syst..

[6] Qiao G., Wang W., Duan W., Zheng F., Sinclair A.J., Chatwin C.R. (2012). Bioimpedance analysis for the characterization of breast cancer cells in suspension. IEEE Trans. Biomed. Eng..

[7] Wi H., Sohal H., McEwan A.L., Woo E.J., Oh T.I. (2014). Multi-frequency electrical impedance tomography system with automatic self-calibration for long-term monitoring. IEEE Trans. Biomed. Circuits Syst..

[8] Jafari H., Soleymani L., Genov R. (2012). 16-channel CMOS impedance spectroscopy DNA analyser with dual-slope multiplying ADCs. IEEE Trans. Biomed. Circuits Syst..

[9] Park J.-Y., Park S.-M. (2009). DNA hybridization sensors based on electrochemical impedance spectroscopy as a detection tool. Sensors.

[10] Manickam A., Johnson C.A., Kavusi S., Hassibi A. (2012). Interface design for CMOS-integrated electrochemical impedance spectroscopy (EIS) biosensors. Sensors.

[11] Yang L., Bashir R. (2008). Electrical/electrochemical impedance for rapid detection of foodborne pathogenic bacteria. Biotechnol. Adv..

[12] Munoz-Berbel X., Vigues N., Jenkins A.T.A., Mas J., Munoz F.J. (2008). Impedimetric approach for quantifying low bacteria concentrations based on the changes produced in the electrode-solution interface during the pre-attachment stage. Biosens. Bioelectron..

[13] Guimera A., Gabriel G., Prats-Alfonso E., Abramova N., Bratov A., Villa R. (2015). Effect of surface conductivity on the sensitivity of interdigitated impedimetric sensors and their design considerations. Sens. Actuators B Chem..

[14] Mallen-Alberdi M., Vigues N., Mas J., Fernandez-Sanchez C., Baldi A. (2016). Impedance spectral fingerprint of E. coli cells on interdigitated electrodes: A new approach for label free and selective detection. Sens. Bio-Sens. Res..

[15] Felice C.J., Valentinuzzi M.E. (1999). Medium and interface components in impedance microbiology. IEEE Trans. Biomed. Eng..

[16] Fernandez-Sanchez C., McNeil C.J., Rawson K., Nilsson O. (2004). Disposable noncompetitive immunosensor for free and total prostate-specific antigen based on capacitance measurement. Anal. Chem..

[17] Ha S., Kim C., Chi Y.M., Akinin A., Maier C., Ueno A., Cauwenberghs G. (2014). Integrated circuits and electrode interfaces for noninvasive physiological monitoring. IEEE Trans. Biomed. Eng..

[18] Ma H., Su Y., Nathan A. (2015). Cell constant studies of bipolar and tetrapolar electrode systems for impedance measurement. Sens. Actuators B Chem..

[19] Felice C.J., Valentinuzzi M.E., Vercellone M.I., Madrid R.E. (1992). Impedance bacteriometry: Medium and interface contributions during bacterial growth. IEEE Trans. Biomed. Eng..

[20] Segura-Quijano F., Sacristan-Riquelme J., Garciaa-Cantonn J., Oses M.T., Baldi A. (2010). Towards fully integrated wireless impedimetric sensors. Sensors.

[21] Song K., Ha U., Park S., Bae J., Yoo H.-J. (2015). An impedance and multi-wavelength near-infrared spectroscopy IC for non-invasive blood glucose estimation. IEEE J. Solid State Circuits.

[22] Manickam A., Chevalier A., McDermott M., Ellington A.D., Hassibi A. (2010). A CMOS electrochemical impedance spectroscopy (EIS) biosensor array. IEEE Trans. Biomed. Circuits Syst..

[23] Ma H., Wallbank R.W.R., Chaji R., Li J., Suzuki Y., Jiggins C., Nathan A. (2013). An impedance-based integrated biosensor for suspended DNA characterization. Sci. Rep..

[24] Hsu C., Jiang H., Venkatesh A.G., Hall D.A. (2015). A hybrid semi-digital transimpedance amplifier with noise cancellation technique for nanopore-based DNA sequencing. IEEE Trans. Biomed. Circuits Syst..

[25] Gao W., Emaminejad S., Nyein H.Y.Y., Challa S., Chen K., Peck A., Javey A. (2016). Fully integrated wearable sensor arrays for multiplexed in situ perspiration analysis. Nature.

[26] Hartov A., Mazzarese R.A., Reiss F.R., Kerner T.E., Osterman K.S., Williams D.B., Paulsen K.D. (2000). A multichannel continuously selectable multifrequency electrical impedance spectroscopy measurement system. IEEE Trans. Bio Med. Eng..

[27] Song K., Ha U., Lee J., Bong K., Yoo H.J. (2014). An 87-mA · min iontophoresis controller IC with dual-mode impedance sensor for patch-type transdermal drug delivery system. IEEE J. Solid State Circuits.

[28] Chi T., Park J.S., Butts J.C., Hookway T.A., Su A., Zhu C., Styczynski M.P., McDevitt T.C., Wang H. (2015). A Multi-Modality CMOS sensor array for cell-based assay and drug screening. IEEE Trans. Biomed. Circuits Syst..

[29] Sun T., Morgan H. (2010). Single-cell microfluidic Impedance cytometry: A review. Microfluid. Nanofluid..

[30] Heidari H., Bonizzoni E., Gatti U., Maloberti F. (2015). A CMOS current-mode magnetic hall sensor with integrated front-end. IEEE Trans. Circuits Syst. I Regul. Pap..

[31] Goldstein B., Kim D., Xu J., Vanderlick T.K., Culurciello E. (2012). CMOS low current measurement system for biomedical applications. IEEE Trans. Biomed. Circuits Syst..

[32] Kim D., Goldstein B., Tang W., Sigworth F.J., Culurciello E. (2013). Noise analysis and performance comparison of low current measurement systems for biomedical applications. IEEE Trans. Biomed. Circuits Syst..

[33] Bennati M., Thei F., Rossi M., Crescentini M., D’Avino G., Baschirotto A., Tartagni M. 20.5 A Sub-pA ΔΣ Current amplifier for single-molecule nanosensors. Proceedings of the 2009 IEEE International Solid-State Circuits Conference—Digest of Technical Papers.

[34] Zhao Y., Zhao J., Wang X., Xia G.M., Qiu A.P., Su Y., Xu Y.P. (2015). A sub-μg bias-instability MEMS oscillating accelerometer with an ultra-low-noise read-out circuit in CMOS. IEEE J. Solid-State Circuits.

[35] Ferrari G., Gozzini F., Molari A., Sampietro M. (2009). Transimpedance amplifier for high sensitivity current measurements on nanodevices. IEEE J. Solid State Circuits.

[36] Crescentini M., Bennati M., Carminati M., Tartagni M. (2014). Noise limits of CMOS current interfaces for biosensors: A Review. IEEE Trans. Biomed. Circuits Syst..

[37] De Langen K.J., Huijsing J.H. (1998). Compact low-voltage power-efficient operational amplifier cells for VLSI. IEEE J. Solid State Circuits.

[38] Hsu C.-C., Wu J.T. (2003). A highly linear 125-MHz CMOS switched-resistor programmable-gain amplifier. IEEE J. Solid State Circuits.

[39] Chai K.T.C., Davies J.H., Cumming D.R.S. (2007). Electrical impedance tomography for sensing with integrated microelectrodes on a CMOS microchip. Sens. Actuators B Chem..

[40] Valente V., Jiang D., Demosthenous A. Design of a wideband CMOS impedance spectroscopy ASIC analog front-end for multichannel biosensor interfaces. Proceedings of the IEEE 2015 Engineering in Medicine and Biology Conference (EMBC).

